# Supplementary material for: From sequence to enzyme mechanism using multi-label machine learning
Source: BMC Bioinformatics. 2014 May 19;15:150. doi: 10.1186/1471-2105-15-150 (PMC4229970; doi:10.1186/1471-2105-15-150)
Supplement: Additional file 2 — Java code of ml2db. Additional file ml2db_code.tar.gz contains the Java source code to run the multi-label machine learning experiments and save the results to database. The code’s Javadoc is included. [file 1471-2105-15-150-S2.zip › additional file 2/ml2db/ml2db/doc/mulan/evaluation/loss/BipartitionLossFunctionBase.html]

BipartitionLossFunctionBase


JavaScript is disabled on your browser.


- Overview
- Package
- Class
- Use
- Tree
- Deprecated
- Index
- Help

- Prev Class
- Next Class

- Frames
- No Frames

- All Classes

- Summary:
- Nested |
- Field |
- Constr |
- Method

- Detail:
- Field |
- Constr |
- Method


mulan.evaluation.loss

## Class BipartitionLossFunctionBase

- java.lang.Object
- - mulan.evaluation.loss.BipartitionLossFunctionBase

- All Implemented Interfaces:
  :   java.io.Serializable, BipartitionLossFunction, MultiLabelLossFunction

  Direct Known Subclasses:
  :   HammingLoss, HierarchicalLoss

  ---

    

  ```
  public abstract class BipartitionLossFunctionBase
  extends java.lang.Object
  implements BipartitionLossFunction, java.io.Serializable
  ```

  Base class for bipartition loss functions

  Version:
  :   2010.11.10

  Author:
  :   GrigoriosTsoumakas

  See Also:
  :   Serialized Form

- - ### Constructor Summary

    Constructors

    Constructor and Description

    `BipartitionLossFunctionBase()`
  - ### Method Summary

    Methods

    | Modifier and Type | Method and Description |
    | --- | --- |
    | `abstract double` | `computeLoss(boolean[] bipartition, boolean[] groundTruth)` Computes the bipartition loss function |
    | `double` | `computeLoss(MultiLabelOutput prediction, boolean[] groundTruth)` Computes the loss function |

    - ### Methods inherited from class java.lang.Object

      `equals, getClass, hashCode, notify, notifyAll, toString, wait, wait, wait`
    - ### Methods inherited from interface mulan.evaluation.loss.MultiLabelLossFunction

      `getName`

- - ### Constructor Detail


    - #### BipartitionLossFunctionBase

      ```
      public BipartitionLossFunctionBase()
      ```
  - ### Method Detail


    - #### computeLoss

      ```
      public final double computeLoss(MultiLabelOutput prediction,
                       boolean[] groundTruth)
      ```

      **Description copied from interface: `MultiLabelLossFunction`**

      Computes the loss function

      **Specified by:**
      :   `computeLoss` in interface `MultiLabelLossFunction`

      Parameters:
      :   `prediction` - the prediction of the learner for an example
      :   `groundTruth` - the ground truth of the example

      Returns:
      :   the value of the loss function


    - #### computeLoss

      ```
      public abstract double computeLoss(boolean[] bipartition,
                       boolean[] groundTruth)
      ```

      **Description copied from interface: `BipartitionLossFunction`**

      Computes the bipartition loss function

      **Specified by:**
      :   `computeLoss` in interface `BipartitionLossFunction`

      Parameters:
      :   `bipartition` - the biprtition of the learner for an example
      :   `groundTruth` - the ground truth of the example

      Returns:
      :   the value of the loss function


- Overview
- Package
- Class
- Use
- Tree
- Deprecated
- Index
- Help

- Prev Class
- Next Class

- Frames
- No Frames

- All Classes

- Summary:
- Nested |
- Field |
- Constr |
- Method

- Detail:
- Field |
- Constr |
- Method
